# How Important Is Diabetes as a Risk Factor for Cardiovascular and Other Diseases in Older Adults?

**DOI:** 10.1371/journal.pmed.0030424

**Published:** 2006-10-17

**Authors:** Andre Pascal Kengne, Anushka Patel

## Abstract

Patel and Kengne discuss a new study in*PLoS Medicine* which found a 2-fold increased risk of cardiovascular death associated with diabetes in people over 65 years old.

It is well established that diabetes mellitus is associated with adverse health outcomes. Data from general population cohorts indicate a 2- to 3-fold increase in cardiovascular risks and about a 50 percent increase in the risks of non-cardiovascular mortality associated with this condition [1–3]. These associations appear largely consistent across populations in different regions of the world [3].

There is some evidence that diabetes may be a more important determinant of cardiovascular risk for women than men [4]. However, the relative effects of diabetes on vascular and other diseases among older, compared with younger, individuals is less certain. Heterogeneity by age in the association between diabetes and cardiovascular disease has been reported, with a consistently weaker association observed among older individuals [3,5]. Given this possible age-dependency in the epidemiological associations, and the frequent observation that cardiovascular risk factors are often managed less aggressively in older people than in younger people [6], a better understanding of the relationship between diabetes and disease-specific causes of death among older people is important.

## A New Cohort Study

In this regard, the data provided by Kronmal and colleagues in *PLoS Medicine* [7] make an important contribution to our knowledge of morbidity and mortality associated with diabetes mellitus in older adults. The researchers evaluated a randomly selected cohort of 5,372 people aged 65 years and over, of whom 8.8 percent were known to have a diagnosis of diabetes and were treated with oral hypoglycaemic agents and/or insulin at baseline. After an average of 11.1 years follow-up, over 40 percent of the cohort had died, with approximately 50 to 60 percent of these deaths attributed to cardiovascular causes. Compared to those without diabetes, and after adjustment for a wide range of covariates, individuals with known, treated diabetes had an estimated excess risk of death ranging between approximately 30 and 100 percent, depending on whether or not they were treated with insulin. For cardiovascular mortality, there was a 2-fold increased risk associated with diabetes.

Cardiovascular risk factors are often managed less aggressively in older people than in younger people.

However, it is likely that the reported hazard ratios in this study underestimate the true strength of associations between diabetes and cause-specific mortality, for two important reasons. First, participants without a diagnosis of diabetes, but with a fasting blood glucose level consistent with this diagnosis, were considered not to have diabetes. Second, for cardiovascular mortality, inclusion of subclinical atherosclerosis as a covariate probably represents over-adjustment.

Nonetheless, these data provide reliable evidence that diabetes is an important adverse risk factor among older adults, with estimates of the strength of the associations comparable to published data from younger cohorts. Furthermore, in this population of (albeit limited) age range, no age interactions in any of these associations were observed.

Kronmal and colleagues also report on analyses suggesting that the relative risks of non-cardiovascular disease mortality associated with diabetes, particularly with respect to death due to infectious or renal causes, were significantly greater among individuals treated with insulin compared with those receiving oral hypoglycaemic agents alone. They further report that women with diabetes on insulin had a particularly high risk of death compared with women without diabetes. These findings are interesting but, in relation to any implication that insulin use may lead to poorer outcomes, can only be considered hypothesis-generating.

One of the strengths of this new study is the availability of data relating to a large number of potentially confounding variables at baseline, and adjustment for these factors has been appropriately made. However, as is always the case with observational data, one cannot account for unmeasured (e.g. duration of diabetes [8]) or unknown risk factors, and residual confounding remains a highly plausible explanation for these findings.

## Clinical Implications

So what are the implications of the results of this study for clinical practice? Primarily, these data confirm that older adults with diabetes are at very high *absolute* risk of death from cardiovascular causes (four to five percent per year). Thus, strategies aimed at reducing these risks should be aggressively pursued among such individuals, wherever possible.

Fortunately, a range of preventive treatments of proven efficacy are at our disposal, including blood pressure lowering [9] and the use of statins [10]. Intensive glucose lowering in type 2 diabetes has been shown to reduce microvascular (retinal and renal) events. However, the balance of risks and benefits of lowering haemoglobin A1c levels below seven percent (as recommended by many current guidelines), particularly with respect to macrovascular events such as myocardial infarction and stroke, remains uncertain. At least two large-scale randomised clinical trials evaluating this question are ongoing, one of which has no upper age restriction [11] while the other includes participants aged up to 80 years at randomisation [12]. Importantly, to reach such targets for intensive glucose lowering, insulin therapy will be frequently required. Should the trials demonstrate that the benefits of intensive glucose lowering outweigh the risks, these data, rather than observational data suggesting possible harm associated with the use of insulin, should take precedence in guiding clinical practice.
